# The BRACELET Study: surveys of mortality in UK neonatal and paediatric intensive care trials

**DOI:** 10.1186/1745-6215-11-65

**Published:** 2010-05-26

**Authors:** Claire Snowdon, Sheila E Harvey, Peter Brocklehurst, Robert C Tasker, Martin P Ward Platt, Elizabeth Allen, Diana Elbourne

**Affiliations:** 1 Medical Statistics Unit, London School of Hygiene and Tropical Medicine, Keppel Street, London WC1E 7HT, UK; 2 Centre for Family Research, University of Cambridge, Free School Lane, Cambridge, CB2 3RF, UK; 3 National Perinatal Epidemiology Unit, University of Oxford, Old Road Campus, Headington, Oxford OX3 7LF, UK; 4 University of Cambridge Clinical School, Department of Paediatrics, Box 116, Addenbrooke's Hospital, Hills Road, Cambridge CB2 2QQ, UK; 5 Newcastle Neonatal Service, Royal Victoria Infirmary, Newcastle, Ward 35, Royal Victoria Infirmary, Newcastle upon Tyne, NE1 4LP, UK

## Abstract

**Background:**

The subject of death and bereavement in the context of randomised controlled trials in neonatal or paediatric intensive care is under-researched. The objectives of this phase of the Bereavement and RAndomised ControlLEd Trials (BRACELET) Study were to determine trial activity in UK neonatal and paediatric intensive care (2002-06); numbers of deaths before hospital discharge; and variation in mortality across intensive care units and trials and to determine whether bereavement support policies were available within trials. These are essential prerequisites to considering the implications of future policies and practice subsequent to bereavement following a child's enrolment in a trial.

**Methods:**

The units survey involved neonatal units providing level 2 or 3 care, and paediatric units providing level II care or above; the trials survey involved trials where allocation was randomized and interventions were delivered to intensive care patients, or to parents but designed to affect patient outcomes.

**Results:**

Information was available from 191/220 (87%) neonatal units (149 level 2 or 3 care); and 28/32 (88%) paediatric units. 90/177 (51%) eligible responding units participated in one or more trial (76 neonatal, 14 paediatric) and 54 neonatal units and 6 paediatric units witnessed at least one death. 50 trials were identified (36 neonatal, 14 paediatric). 3,137 babies were enrolled in neonatal trials, 210 children in paediatric trials. Deaths ranged 0-278 (median [IQR interquartile range] 2 [1, 14.5]) per neonatal trial, 0-4 (median [IQR] 1 [0, 2.5]) per paediatric trial. 534 (16%) participants died post-enrolment: 522 (17%) in neonatal trials, 12 (6%) in paediatric trials. Trial participants ranged 1-236 (median [IQR] 21.5 [8, 39.8]) per neonatal unit, 1-53 (median [IQR] 11.5 [2.3, 33.8]) per paediatric unit. Deaths ranged 0-37 (median [IQR] 3.5 [0.3, 8.8]) per neonatal unit, 0-7 (median [IQR] 0.5 [0, 1.8]) per paediatric unit. Three trials had a formal policy for responding to bereavement.

**Conclusions:**

A substantial number of deaths after trial enrolment were identified, distributed over many trials and units. Few trial teams had responses to bereavement in place. Those with the largest numbers of deaths might be best placed to collaborate in developing and assessing responses to bereavement.

## Background

The current emphasis on the need for good evidence to guide care [1,2], and the establishment of the UK Medicines for Children Research Network (MCRN) to encourage and facilitate paediatric research, suggest that increasing numbers of children will be enrolled into randomised controlled trials. This includes extremely sick children in neonatal and paediatric intensive care units, of whom a proportion will die before discharge home. The subject of death and bereavement in the context of trials is, however, under-researched.

It is not known how many participants are enrolled in this setting, or how many survive or die. The parents of those who go on to die subsequent to trial enrolment may have a range of information and support needs and preferences but these have not yet been adequately described and explored. We do not know whether bereaved parents might wish to have further contact with a trial, and what services, if any, they might wish to access; we do not know what approaches clinicians and trial teams might feel able to offer.

An essential prerequisite to considering bereavement and trials is to ascertain the magnitude and distribution of post-trial mortality. This study therefore aimed to determine:

1. trial activity in UK neonatal and paediatric units;

2. the number and proportion of deaths among babies and children participating in trials in intensive care;

3. variation in mortality across units, and across trials

4. whether any provision is made for bereavement within trials

## Methods

**Figure 1:**
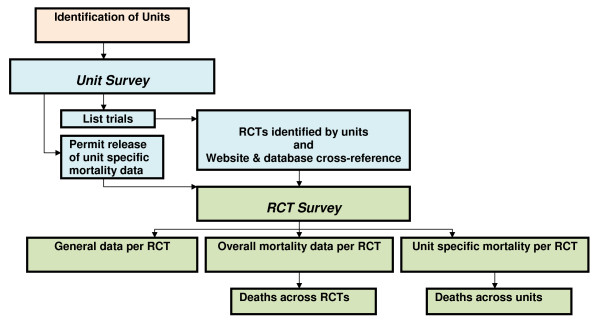
**Structure of BRACELET Study Surveys**.

Although new trials are increasingly being registered, especially those involving new medical products, there is no single repository of trials through which all trials conducted in the UK over specified time periods and particular specialties can be identified. The **B**ereavement and **RA**ndomised **C**ontrol**LE**d **T**rials (BRACELET) Study therefore required two linked surveys to achieve its objectives; the first survey involved neonatal and paediatric units to identify trials conducted in the UK in 2002-2006; the second survey involved trials to collate data on deaths across trials and across their collaborating neonatal and paediatric units (Figure 1).

### Unit survey

The unit survey aimed to identify all trials open to recruitment in the UK from 1 January 2002 to 31 December 2006. Data were requested from all neonatal units providing care designated as Level 2 (high dependency and some short-term intensive care) or Level 3 (whole range of medical care but not necessarily specialist services such as surgery) [3], and all paediatric units with a paediatric intensivist in post which provide at least Level II intensive care (1:1 nurse:child ratio providing care for those requiring continuous nursing supervision, usually intubated and ventilated, or unstable non-intubated or recently extubated) [4].

Two hundred and twenty neonatal unitsand 32 paediatric units were identified through a process of cross-checking multiple sources [3-9]. Units were contacted by post but questionnaires were also made available on the BRACELET website http://www.bracelet-study.org.uk. One hundred and forty nine neonatal units reported their designated level of care as Level 2 or 3 and were eligible to participate in the study. Representatives at these 149 neonatal units, and at the 32 Level III paediatric units [7,9] were asked to complete a questionnaire in April 2007. This asked respondents to list all trials open to recruitment in their unit in 2002-2006. The clinical lead for each unit was asked to permit the trial coordinating team for each trial to which they had recruited to release that unit's recruitment and mortality data to the BRACELET Study. Two reminders were sent via email or mail. Nurse Practitioners from MCRN made additional follow up contact where appropriate. Opportunistic and direct contact between study members and units also served as reminders. Data collection was concluded in May 2008.

### Trials survey

The unit survey generated a list of trials which was supplemented by searches of specialised websites [8-10]. Many of the trials were also identified through other sources such as the UK Dept Health National Research Register https://portal.nihr.ac.uk/Pages/NRRArchive.aspx, PubMed http://www.ncbi.nlm.nih.gov/pubmed, PICS website http://www.ukpics.org, and the European Society of Paediatric and Neonatal Intensive Care website http://www.espnic.de.

Trials were eligible for the survey if: allocation was randomised; enrolment took place during the five year study period; parental informed consent was required; and the intervention was delivered to babies or children within ICUs or delivered by, or under the auspices of, a neonatologist or paediatric intensivist leading to ICU admission for ongoing care, or the intervention was delivered to parents but designed to affect outcomes for babies or children.

For each eligible trial, the chief investigator, trial manager or other appropriate contact was asked to complete an emailed questionnaire. Questionnaires were also made available on the BRACELET website http://www.bracelet-study.org.uk/index.php?page=previous-research---phase-1 were followed up by telephone, direct contact and the assistance of MRCN, if necessary. The information received was supplemented by data from published papers, relevant websites and personal communication. Three types of data were generated, for the five year study period only: *general data *about trials (outcome measures, participating units, numbers enrolled); *overall mortality data *(UK mortality per trial before discharge from hospital) and *unit-specific mortality data *(deaths per unit per trial before discharge from hospital). Chief investigators, trial managers or other appropriate contacts were also asked to provide copies of the trial protocol and parent information leaflets for their trial.

### Analysis

Descriptive data are presented as proportions and ranges, as appropriate. Analysis used the statistical package Stata 10 (StataCorp, College Station, Texas, USA). Variations in the denominators for some of the numbers reported in the results reflect different response rates for the unit survey and the trials survey, and incomplete release of mortality data by some units and some trials.

### Ethics

Ethics committee approval was not required for this phase of the BRACELET Study.

## Results

### Response rates

#### Unit survey

Questionnaires were sent to 220 neonatal units; 191 (86.8%) responded, of which 149 were eligible units (82 providing Level 2 care and 67 Level 3). Questionnaires were also completed by 28 (87.5%) of the 32 Level II paediatric units surveyed.

#### Trials survey

The unit survey and associated searches identified 50 trials (36 neonatal and 14 paediatric trials). Some general data were obtained for 43 trials (32 neonatal, 11 paediatric). Overall UK mortality data were released for 37 trials (28 neonatal, 9 paediatric). Unit-specific mortality data were released for 33 trials (24 neonatal, 9 paediatric) for those ICUs which had permitted release of their data to the study in the unit survey.

### Survey findings

The unit survey indicated that overall half of the ICUs enrolled one or more participants in one or more trials during the five year study period (76/149 neonatal units, 14/28 paediatric units) (Table 1).

**Table 1:** NICU and PICU participation in RCTs (based on respondents to unit surveys)

No. RCTs	NICUs	PICUs
	
	Total, n = 149 n (%)	n = 28 n (%)
0	73 (49.0)	14 (50.0)
1	31 (20.8)	8 (28.6)
2	19 (12.8)	3 (10.7)
3	13 (8.7)	1 (3.6)
≥4	13 (8.7)*	2 (7.1)**
**≥ 1 RCT**	**76 (51.0)**	**14 (50.0)**

* 10 × 4, one each 5, 6, and 7

**One 4 and one 7

A minority of the responding Level 2 neonatal units (N = 27, 32.9%) and the majority of the responding Level 3 neonatal units contributed to a trial (N = 49 (73.1%). Nine (13.4%) of the Level 3 neonatal units ran their own single centre trials but none of the Level 2 neonatal units did so. Five of the 14 responding paediatric units (17.9%) ran single centre trials.

#### General data

Of the 76 neonatal units which enrolled to a trial, 72 provided details of the number of babies enrolled. A total of 3117 babies were enrolled by these neonatal units into the 29 neonatal trials for which some enrolment data for the five year study period were available. The number of babies enrolled per neonatal unit ranged 1-236 (median [IQR] 21.5 [8, 39.8]). An additional 20 babies were recruited into two multicentre neonatal trials by two paediatric units, bringing the total enrolled in neonatal trials to 3137 babies. Of these 480 (15.3%) were recruited into single centre trials and 2657 (84.7%) into multicentre trials (UK and international) (Table 2).

**Table 2:** Babies and children enrolled 2002-2006 by type of trial and by enrolling unit (neonatal or paediatric)

	No. enrolled from neonatal units	No. enrolled from paediatric units	Total no. enrolled
**NEONATAL TRIALS**			

**No. of trials**	29	2	29*

**No. of units**	36	2	38

**No. of babies enrolled**	3117	20	3137

**No. of babies enrolled per recruiting unit; Median [IQR]**	1-236 21.5 [8, 39.8]	4 and 16**	1-236 20 [7.8, 39.3]

**No. of babies enrolled per trial;** **Median [IQR]**	1-1322 40 [13.5, 104]	4 and16**	5-1326 40 [14.5, 104]

			

**PAEDIATRIC TRIALS**			

**No. of trials**		9	9

**No. of units**		11	11

**No. of children enrolled**		210	210

**No. of children enrolled per recruiting unit;** **Median [IQR]**		1-53 11.5 [2.3, 33.8]	1-53 11.5 [2.3, 33.8]

**No. of children enrolled per trial;** **Median [IQR]**		2-53 10.5 [6, 39.3]	2-53 10.5 [6, 39.3]

			

**ALL NEONATAL/PAEDIATRIC TRIALS**			

**No. of babies/children enrolled**	**3117**	**230**	**3347**

**No. of trials**	29	11	38*

*includes two neonatal trials which recruited from both neonatal and paediatric units

** no median and IQR as only two trials

Of the 14 paediatric units that enrolled into a paediatric trial, 11 provided details of the number enrolled. A total of 210 children were enrolled by these paediatric units into 9 paediatric trials for which some enrolment data for the five year study period were available. The number of children enrolled per paediatric unit into paediatric trials ranged 1-53 (median [IQR] 7 [2], 34). Of these 94 (44.8%) were enrolled into single centre trials and 116 (55.2%) to multicentre trials (all of which were international) (Table 2).

#### Overall mortality data

Overall mortality data were available for 28 neonatal and 9 paediatric trials (Table 3). In total, 534/3288 (16.2%) children died following enrolment in these 37 trials.

**Table 3:** Hospital survivors and non-survivors overall by type of RCT - UK totals 2002-2006 (overall mortality data)

NEONATAL TRIALS (n = 28)	
No. of babies enrolled	3088

No. of babies outcome unknown	2

No. of babies survived	2564

**No. of babies died**	**522**
Mortality rate % (based on known outcomes)	16.9

	

**PAEDIATRIC TRIALS (n = 9)**	

No. of children enrolled	200

No. of children outcome unknown	0

No. of children survived	188

**No. of children died**	**12**

Mortality rate % (based on known outcomes)	6.0

	

**NEONATAL and PAEDIATRIC TRIALS (n = 37)**	

No. of babies/children enrolled	3288

No. of babies/children outcome unknown	2

No. of babies/children survived	2752

**No. of babies/children died**	**534**

Mortality rate % (based on known outcomes)	16.2

**Figure 2:**
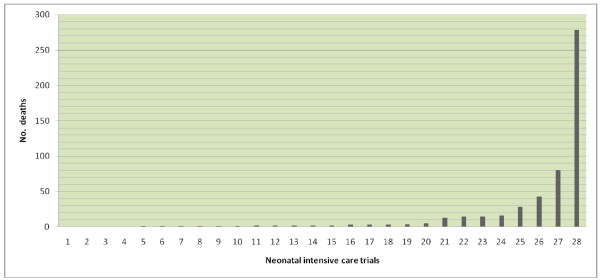
**Variation in numbers of deaths across neonatal trials**.

The 28 neonatal trials enrolled 3,088 babies, of whom 522 (16.9%) died. The number of deaths per neonatal trial ranged 0-278 (median [IQR] 2 [1, 14.5]) (Figure 2). Of the 28 neonatal trials, 24 had at least one death. The highest mortality rate amongst these trials was 29% (80 deaths). Most reported small numbers of deaths (only 8 trials reported >10). The majority of deaths, 429/522 (82.2%), occurred in four trials, three of which were multicentre (N = 278 + 80 + 43 and one single centre (N = 28). Single centre trials reported fewer deaths and a lower death rate (47/480 9.8%) than multicentre trials (475/2608 18.2%).

In the nine paediatric trials for which mortality data were available, 12 (6.0%) out of 200 children died following enrolment into a trial. Six of the 9 trials had a least one death with the number of deaths ranging 0-4. Very few deaths occurred in single centre paediatric trials (2/94 2.1%) compared to those in the neonatal single centre trials (47/480 9.8%) and the paediatric multicentre trials 10/106 (9.4%).

#### Unit-specific mortality data

**Figure 3:**
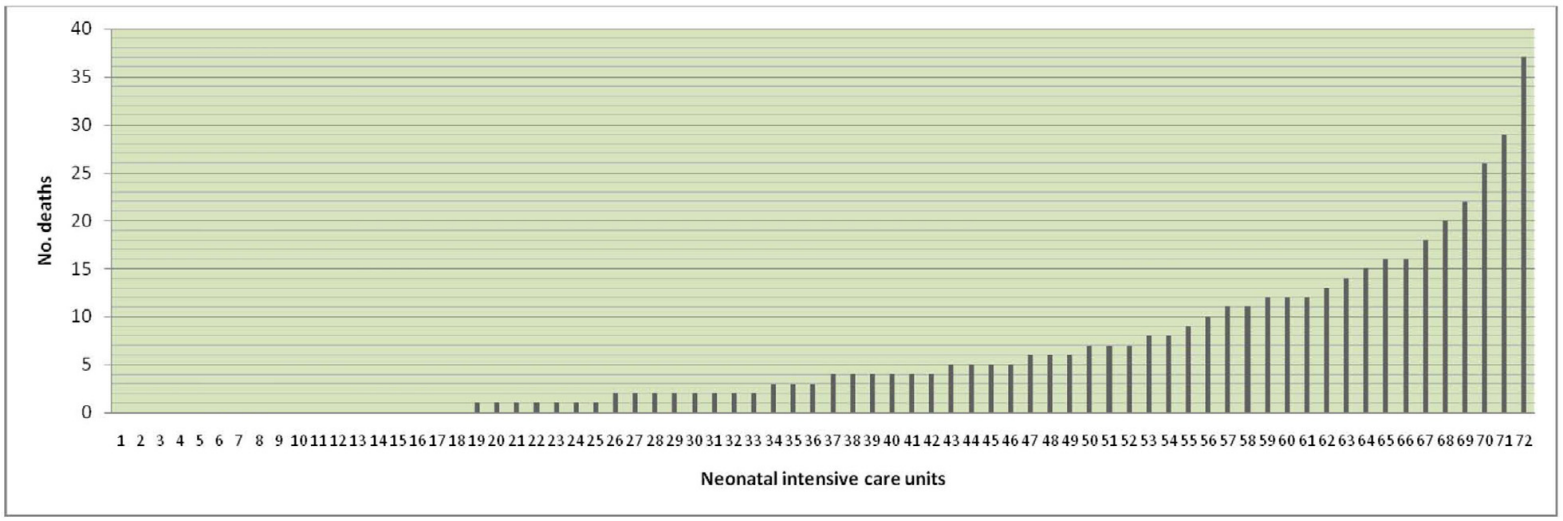
**Variation in numbers of deaths across neonatal units**.

Data on 434 deaths were released by 24 neonatal trials for 72 neonatal units with the permission of the neonatal units in question. The number of deaths per neonatal unit ranged 0-37 (median [IQR] 3.5 [0.3, 8.8]) (Figure 3). Whilst 54 neonatal units saw at least one death, more than half (42/72 58.3%) saw fewer than five deaths over this five year period (Table 4). Five Level 3 neonatal units had larger numbers (N = 37, 29, 26, 22 and 20) and 30.9% of all deaths recorded by the units occurred in these five neonatal units. In around half of the units, the proportion of children who died following trial enrolment was 20% or more (Table 5).

**Table 4:** Numbers of deaths in NICUs and PICUs following enrolment into a trial 2002-2006 (unit-specific mortality data)

No. of deaths	NEONATAL INTENSIVE CARE UNITS	PAEDIATRIC INTENSIVE CARE UNITS
	No. (%) of Units (n = 72)	No. of Units (n = 12*)
None	18 (25)	6

1-4	24 (33)	5

5-9	13 (18)	1

10-14	8 (11)	0

15-19	4 (6)	0

≥20	5 (7)	0

Total no. of deaths in these units	434	14

**No. (%) of units seeing at least one death**	**54 (75)**	**6 (43)**

**Table 5:** Proportion of deaths in NICUs and PICUs following enrolment into a trial 2002-2006 (unit-specific mortality data)

Proportion of deaths	NEONATAL INTENSIVE CARE UNITS	PAEDIATRIC INTENSIVE CARE UNITS
	**No. (%) of Units (n = 72)**	No. of Units (n = 12*)

0	18 (25)	6

0-0.1	10 (14)	3

0.1-0.2	14 (19)	3

0.2-0.3	16 (22)	

0.3-0.4	7 (10)	

0.4-0.5	5 (7)	

≥0.5	2 (3)	

* No mortality information was available for two of the 14 Units

Nine paediatric trials released unit specific mortality data for 14 paediatric units. The number of deaths per paediatric unit ranged 0-7(median [IQR] 0.5 [0, 1.8]), with 6 paediatric units witnessing at least one death (Table 4). In all these units, the proportion of children who died following trial enrolment was under 20% (Table 5).

### Trials survey - Practices in relation to bereavement care

Of the 50 RCTs, investigators for just over half (n = 27) provided a copy of the full trial protocol. None of the protocols documented a policy relating to the care of parents bereaved following enrolment of their child into the RCT.

Parent information leaflets were provided for 29 of the 50 trials. Two NIC trials (one multicentre and one UK-led international trial) provided a leaflet specifically for bereaved parents, expressing condolences, thanking them for their contribution and offering information about the trial. Details of support organisations were also given in the leaflets.

In one single-centre NIC trial the investigator reported a different approach. Three deaths occurred following enrolment into this trial and the investigator sent a personalised letter to each set of parents to thank them for allowing their child to participate and to offer contact should they wish to discuss the trial or the continued use for their child's data in the trial.

## Discussion

The BRACELET Study is the first to investigate randomised controlled trial activity in UK neonatal units and paediatric units, to report the numbers of babies and children enrolled into trials, and to determine the extent and distribution of mortality involved. An important strength of this study is the high response rates achieved. Several evidence-based strategies were used to maximise responses [11]. We are confident that all units were identified, and the comprehensive process of searching relevant research databases and websites as well as surveying these units is likely to have identified most of the trials recruiting in the UK. The establishment of mandatory trial registration will facilitate this process for future studies. There are, however, clear limitations to the study which relate to its narrow focus on mortality figures; in this regard the data raise rather than answer questions about bereavement in this context.

The study shows that in a five year period, over 3000 babies and children were enrolled into paediatric and neonatal intensive care trials and 16% died, predominantly in the neonatal context. With over 500 deaths reported we suggest that a substantial number of bereaved parents, clinicians and trialists have encountered deaths among trial participants. We would also suggest that this is an underestimate as the BRACELET study focused only on deaths up to discharge from hospital; post-discharge deaths were not included. Other adverse outcomes for parents and families, such as disability and loss of quality of life in surviving babies are also important but were beyond the remit of the study.

As further trials are initiated and accrue more participants, the population of parents bereaved after agreeing to enroll their child in a trial will accumulate; it is already sufficiently sizeable to warrant attention, but whether and how to respond to this population are complex questions. Provision for bereavement is often made within clinical centres but this body of parents, with potentially diverse experiences and needs, is largely scattered across a number of recruiting clinical centres; most deaths occurred as relatively isolated cases and the majority of centres witnessed small numbers of deaths per year. In the paediatric context where few deaths occurred, only one ICU reported more than one death. This is likely to make it difficult for many of the clinical centres to develop, assess and sustain specialised responses to post-trial bereavement themselves.

The patterns of mortality revealed by the BRACELET Study also suggest, however, that there were pockets of neonatal units and neonatal trials with substantial numbers of deaths. Five particularly research active Level 3 neonatal units saw 20 or more deaths each in the study period, and together they saw over a quarter of all reported deaths. In general, large ICUs draw upon well developed bereavement services [12], and research-active centres such as these may be appropriate candidates to develop and assess dedicated *trial-related *bereavement practices.

The vast majority of deaths represented in the BRACELET Study, also occurred in only four trials. In trials where a substantial number of deaths is anticipated, it may be possible to develop and assess trial-related bereavement practices.

What form those practices might take is unclear. They may range from development of formal practices and supporting literature to a more simple policy of offering parents the opportunity to discuss a trial if they so wish.

Parents have not yet been asked about any support and information needs that they might have. Their preferences are likely to be varied and may include the wish for no further contact. It is however possible that some options that parents might appreciate, for instance access to specialised forms of support, may be beyond the capacity and expertise of current routine bereavement services, even in the larger centres, and may be difficult for trial teams to implement.

The BRACELET Study showed that three trials had already developed a response to bereavement such as preparing a bereavement leaflet for use in clinical centres or sending condolence letters directly to parents (for an example leaflet see http://www.npeu.ox.ac.uk/downloads/nest/NEST-Bereavement-Leaflet.pdf). Personal communications have revealed that some trials offer bereaved parents the option of receiving trial newsletters and results; some make a considered choice not to contact bereaved parents at all subsequent to a death.

To our knowledge, none of these policies have been subject to empirical evaluation, although descriptive accounts such as Strohm's report of a trial-related web-based message board for all parents of babies recruited to a trial, including those who are bereaved [13], are helpful additions to the literature. Further reflection would be of value to future trials where deaths are likely.

The BRACELET Study has demonstrated that bereavement occurs in relation to trials of any size and type and with a range of clinical foci. The four trials which reported the majority of deaths in the five year period assessed very different interventions, from routine care practices to potentially life-saving technologies. They involved very different populations and were conducted in single centre, multicentre and international contexts. This suggests that bereavement in a trial context may be an issue of broad relevance in specialties such as intensive care, and that it could be particularly appropriate for large trials, or trials focusing on high risk situations, to plan for and assess their approach to bereavement with substantial research populations.

Trials are complex, highly collaborative endeavors between recruiting clinical centres and trial teams, groups which may feel a shared interest in and responsibility for parents bereaved in trials. Their collaboration might be exploited to good effect if experts within these fields take collective responsibility for the potential needs of the population identified here. If those trials and clinical centres with the greatest experience of post-trial bereavement develop effective approaches to care for and support bereaved parents, other smaller trials and centres may draw upon their recommendations and follow their lead. Even in the paediatric context where deaths occurred infrequently, individual trials may still involve severely compromised populations and so find that post-trial bereavement care is a salient issue.

It is, however, important that recommendations in this novel area should from an early stage be based on sound empirical evidence which draws upon views of *all *relevant parties with their potentially different perspectives and insights. Clinical teams often recruit to a number of trials concurrently and see bereaved parents in a variety of circumstances; they may be best placed to consider the broad range of bereavement-related issues that might occur in clinical contexts. Trial teams by comparison consider parents in the relatively more uniform circumstances set by the eligibility criteria for their particular trial; they may be best placed to consider bereavement practices which are tailored to fit the population and circumstances of a given trial. Research in this area is sensitive but it is essential that bereaved parents should also be consulted. Studies have demonstrated that bereavement-related research is feasible [14-20], and suggest that bereaved parents might be willing and helpful participants on this challenging and sensitive subject.

The task ahead is for those with relevant insight and expertise, to collaborate to find a range of approaches which are sensitive to the variety of parents seen by clinicians and applicable and adaptable to the specific circumstances addressed in individual trials. As a first step in this process the BRACELET Study includes a second qualitative component which aims to explore death, dying and bereavement in the context of neonatal RCTs from the perspectives of trial team members, clinicians and bereaved parents.

## Competing interests

The authors declare that they have no competing interests.

## Authors' contributions

The BRACELET Study was devised and designed by CS, DE, PB, RT and MWP. Data were mainly collected by SH and CS. All authors had access to the data in the study and can take responsibility for the integrity of the data and the accuracy of the data analysis. An initial draft of the paper was prepared by SH and CS and revised by all authors. EA undertook supplementary analyses. CS and DE prepared the final version of the paper. All authors read and approved the final manuscript.

## References

[B1] McIntosh N Bates P Brykczynska G Dunstan G Goldman A Harvey D Larcher V McCrae D McKinnon A Patton M Saunders J Shelley P Guidelines for the ethical conduct of medical research involving children. Royal College of Paediatrics, Child Health: Ethics Advisory Committee Arch Dis Child 2000 82 2 177 82 10.1136/adc.82.2.177 10648379 PMC1718211

[B2] Medical Research Council Medical Research involving children 2004 http://www.mrc.ac.uk/Utilities/Documentrecord/index.htm?d=MRC002430 accessed 20 May 2010

[B3] British Association of Perinatal Medicine Standards for hospitals providing neonatal intensive care 2001 Second

[B4] Paediatric Intensive Care Society 2 Standards of care London PICS http://careers.bmj.com/careers/advice/view-article.html?id=2645 accessed 18 August 2008

[B5] Redshaw M Hamilton K A survey of current neonatal unit organisation and policy National Perinatal Epidemiology Unit 2005 http://www.npeu.ox.ac.uk/downloads/reports/bliss-final-report.pdf accessed 18 August 2008

[B6] Acolet D Jelphs K Davidson D Peck E Clemens F Houston R Weindling M Lavis J Elbourne D The BLISS cluster randomised controlled trial of the effect of 'active dissemination of information' on standards of care for premature babies in England (BEADI) study protocol [ISRCTN89683698] Implement Sci 2007 2 33 10.1186/1748-5908-2-33 17922901 PMC2117010

[B7] Directory of Critical Care CMA Medical Data 2006

[B8] British Association of Perinatal Medicine http://www.bapm.org accessed 18 August 2008

[B9] Paediatric Intensive Care Audit Network (PICANet) http://www.picanet.org.uk accessed 18 August 2008

[B10] National Perinatal Epidemiology Unit http://www.npeu.ox.ac.uk accessed 18 August 2008

[B11] McColl E Jacoby A Thomas L Soutter J Bamford C Steen N Thomas R Harvey E Garratt A Bond J Design and use of questionnaires: a review of best practice applicable to surveys of health service staff and patients Health Technol Assess 2001 5 31 1 256 11809125 10.3310/hta5310

[B12] Harvey S Snowdon C Elbourne D Effectiveness of bereavement interventions in neonatal intensive care: A review of the evidence Semin Fetal Neonatal Med 2008 13 5 341 56 10.1016/j.siny.2008.03.011 18514602

[B13] Strohm B The TOBY study parents online message board Journal of Neonatal Nursing 2007 13 107 112 10.1016/j.jnn.2007.01.003

[B14] Brinchmann BS Forde R Nortvedt P What matters to the parents? A qualitative study of parents' experiences with life-and-death decisions concerning their premature infants Nurs Ethics 2002 9 4 388 404 10.1191/0969733002ne523oa 12219402

[B15] Brosig CL Pierucci RL Kupst MJ Leuthner SR Infant end-of-life care: the parents' perspective J Perinatol 2007 27 8 510 6 10.1038/sj.jp.7211755 17443196

[B16] Kavanaugh K Parents' experiences surrounding the death of a newborn whose birth is at the margin of viability J Obstet Gynecol Neonatal Nurs 1997 26 1 43 51 10.1111/j.1552-6909.1997.tb01506.x 9017546

[B17] McHaffie HE Lyon AJ Hume R Deciding on treatment limitation for neonates: the parents' perspective Eur J Pediatr 2001 160 6 339 44 10.1007/PL00008444 11421412

[B18] Snowdon C Elbourne DR Garcia J Perinatal pathology in the context of a clinical trial: attitudes of bereaved parents Arch Dis Child Fetal Neonatal Ed 2004 89 3 F208 11 10.1136/adc.2003.041392 15102721 PMC1721668

[B19] Snowdon C Elbourne DR Garcia J Perinatal pathology in the context of a clinical trial: attitudes of neonatologists and pathologists Arch Dis Child Fetal Neonatal Ed 2004 89 3 F204 7 10.1136/adc.2002.012732 15102720 PMC1721693

[B20] Snowdon C Elbourne D Garcia J Lavender T, Edwards G, Alfirevic Z Embedding a qualitative approach within a quantitative framework: an example in a sensitive setting Demystifying Qualitative Research 2004 London: Quay books

